# Supplementary material for: Learning curve and surgical outcome of robotic assisted colorectal surgery with ERAS program
Source: Sci Rep. 2022 Nov 29;12:20566. doi: 10.1038/s41598-022-24665-w (PMC9709162; doi:10.1038/s41598-022-24665-w)
Supplement: Supplementary file 1 — Supplementary Information 1. [file 41598_2022_24665_MOESM1_ESM.zip › LSC Raw data-Ñ[▒K/111/H_ñw╝╨░O▒Kñσ.pdf]

正式摘要(電子簽章：0000020753 6853)

W52 / W52 英文標題(注意:無英文姓名-請補病人基本檔!) 【讀取】憑證

臺中榮民總醫院

出院病歷摘要

轉入號 : 轉出號 :

[redacted]

(8)流水編號	
(9)入院日期	109/12/23 (2020) 直腸外科 W52 - 155
(10)轉科日期	
(11)出院日期	109/12/29 (2020) 住院天數計 6 日

診斷

- 入院 :colon polyp, cecum
  hypertension
  type 2 diabete mellitus
  asthma
- 出院 :Adenocarcinoma of cecum, TisN0M0, s/p lap right hemicolectomy on 2020/12/24
  hypertension
  type 2 diabete mellitus
  asthma

主訴

- colon polyp was found in health examination

病史

- This 63 years old femalepatient has past history of hypertension, type 2 diabete mellitus and asthma under medical control. She received health examination and colon polyp was found around ileocecal valve. EMR was tried initially but failed. There was no biopsy report. Thus, she was transferred to our hospital for surgical intervention.

體檢發現

-
  - General appearance :
    - easy looking , conscious clear , without cardiopulmonary distress
    - Vital signs自動帶入date 體溫(BT) 脈搏(P) 呼吸(R) 血壓(BP) 身高(BH) 體重(BW) BMI
      20201223 35.9℃ 72次/分 19次/分 120/83mmHg 159.3cm 77.7kg 30.6
  - HEENT :
    - Eyes: no anemic conjunctiva, no icteric sclera, normal EOM
    - ENT: no otorrhea, no rhinorrhea, no erythematous mucosa, no injected tonsil, no deformity
  - Neck: supple, freely movable, trachea midline,no deformity, no engorged jugular vein , no palpable thyroid or lymph node enlargement
  - Chest:
    - Inspection: Grossly normal, symmetric and fully expansion, no accessory muscle used, no operation scar, no spider angiomata, no gynecomastia
    - Palpation: no palpable mass or nodule, no subcutaneous emphysema, no tenderness
    - Asculation: clear breathing sound
  - Heart:
    - Percussion: normal heart size
    - Asculation: regular heart beat, normal S1, S2, no gallop, no murmur
  - Abdomen:
    - Inspection: in
      - flat contour, symetrically, no scar, no stria, no dilated veins, no rashes
    - Asculation: normoactive bowel sound, no abdominal bruits

- palpation: soft, no muscle guarding or rigidity, no local tenderness, no rebounding pain, no palpable mass, no palpable pulsation
- Percussion: no shifting dulllness, no hepatomegaly, no splenomagaly

Peripheral pulsation :	CA	BA	RA	FA	PA	PTA	DPA
Right side :	++	++	++	++	++	++	++/+++
Left side :	++	++	++	++	++	++	++/+++

- Genito-anus: no tenderness or palpable mass lesion or rectal shelf, normal external genitalia, normal anal tone, brown stool

CRS CONDITION

- Lung

手術日期及方法

- 手術日期及方法20201224 Laparoscopic right colectomy and anastomosis

住院治療經過

- After admission, operation with right hemicolectomy was done on 12/24. We let her try water on POD1 and then liquid diet. Penrose drain was removed on 12/29 due to clean and less drain amount. Under the stable condition, she was discharged.

合併症

- nil

一般檢查

- 累積報告 :

DATE	TIME	ALB	TP	BIL.T	BIL.D	ALKP	AST	ALT	LDH	CHOL	TG	HDL-C	LDL	UA	NH3	AMY
1091223	1753		6.1	0.6	0.1		17	19								
1091225	0904							19								

DATE	TIME	WBC	RBC	HGB	HCT	MCV	PLT	BLST	NEUT	BAND	SEG	LYM	MON	EOS	BAS
1091223	1753	6970	4.38	14.2	40.9	93.4	270		56.9			30.8	4.9	6.5	0.9
1091225	0856	11030		15.1			292		83.8			11.7	4.4	0.0	0.1
1091228	0913	6920		14.1	39.2		259		64.4			24.7	5.1	5.5	0.3

DATE	TIME	NA	K	CL	CA	BUN	CREAT	Mg	P	CRP	ESR	PCT	LIPASE	LACTATE
1091223	1753	142	3.1	104	9.1	14	0.58							
1091225	0904	142	3.3			9	0.65							
1091228	0929	140	3.3			7	0.66							

DATE	TIME	GLUCOSE	PAB	TRF	ZN	CU	KETONE	CK	CKMB	PT-P	PT-C	APTT-P	APTT-C	TRO-T
1091223	1753	160								10.7	10.3	25.6		

特殊檢查

病理報告

其它

出院時情況

- 3.改門診治療

出院指示

藥名	劑量	劑量單位	用法	頻次	數量	數量單位	現況	天數	單位
Gaslan tab 40mg	1	TAB	PO	QID	28	TAB	TH	7	天
Norvasc tab 5mg	1	TAB	PO	QD	7	TAB	TH	7	天
Pantoloc tab 40mg	1	TAB	PO	QDAC	7	TAB	TH	7	天

出院計劃

主治醫師_2062F 蔣鋒帆 / 住院醫師_2331F 陳凱祥

正式摘要(電子簽章：000000207536853)

W52 / W52

doctorRank:B
